# The defect challenge of wide-bandgap semiconductors for photovoltaics and beyond

**DOI:** 10.1038/s41467-022-32131-4

**Published:** 2022-08-11

**Authors:** Alex M. Ganose, David O. Scanlon, Aron Walsh, Robert L. Z. Hoye

**Affiliations:** 1 Department of Materials, Imperial College London, Exhibition Road, London, SW7 2AZ UK; 2 Department of Chemistry, University College London, 20 Gordon Street, London, WC1H 0AJ UK; 3 Thomas Young Centre, University College London, Gower Street, London, WC1E 6BT UK

**Keywords:** Solar cells, Energy

## Abstract

The optoelectronic performance of wide-bandgap semiconductors often cannot compete with that of their defect-tolerant small-bandgap counterpart. Here, the authors outline three main challenges to overcome for mitigating the impact of defects in wide-bandgap semiconductors.

Visible-light harvesting materials with bandgaps in the range of 1.6–2.5 eV are gaining increasing significance across a range of emerging technologies, such as indoor and tandem photovoltaics (PVs), but feature inferior optoelectronic performance compared to their small-bandgap (<1.6 eV) counterparts. Charge-carriers tend to have shorter diffusion lengths, along with higher trapping and recombination rates. We outline the critical challenges for achieving defect-tolerant wide-bandgap crystals, and suggest promising routes forward.

## Efficient, low-cost devices through defect tolerance

Defects are ubiquitous in crystalline semiconductors. Managing their influence on electronic properties is critical for high performance across a range of technologies, including PVs, photoelectrochemical cells and radiation detectors. Of particular importance are point defects—including vacancies, anti-sites or interstitials—which lead to the introduction of electronic states that fall within the bandgap (*E*_g_), resulting in irreversible losses in charge-carriers through trapping and non-radiative recombination. Historically, the effects of defects were mitigated by minimising their concentration through careful (and often expensive) fabrication methods, such as the case for Si^1^. More recently, new classes of semiconductors have been discovered that can maintain high light conversion efficiency despite high concentrations of point defects. Such defect tolerance can arise in several ways. Defects can pair together to form electronically benign complexes (e.g., in CuInSe_2_)^2,3^. Defects can also be less harmful if: (i) the dominant traps within the bandgap are energetically close to the valence or conduction band edges (i.e., shallow), or (ii) have low capture cross-sections (see ref. 4 for details). These effects have been found in metal-halide perovskites, and are an important factor behind their rapid achievement of PV efficiencies comparable to those of Si^1,4^, despite being processed at significantly lower temperatures that enable lower levelised cost of energy and CO_2_eq footprint^5^.

## Wide-bandgap challenge

Semiconductors that have both been demonstrated to be defect tolerant and also realised in efficient PV (i.e., CuInSe_2_ and iodide-based perovskites) have only been achieved for materials with <1.6 eV bandgap^1–3^. Wider-bandgap semiconductors, with *E*_g_ in the range of 1.6–2.5 eV, have had less success, but are now becoming increasingly important for a range of clean energy and healthcare applications. These include indoor PV (optimal *E*_g_ of 1.9 eV) to sustainably power the Internet of Things^6^, photoelectrochemical cells (~2 eV) for single-junction solar-to-hydrogen water splitting^7^, X-ray detectors (1.4–2.5 eV) for medical imaging and security^4^, and top-cells (>1.7 eV) for tandem PV with silicon^8^. There are a number of obstacles to overcome. Below, we discuss the critical challenges to achieving defect tolerance in wide-gap semiconductors, and inspiration on how these challenges could be overcome.

### Harder dopability

**Fig. 1:**
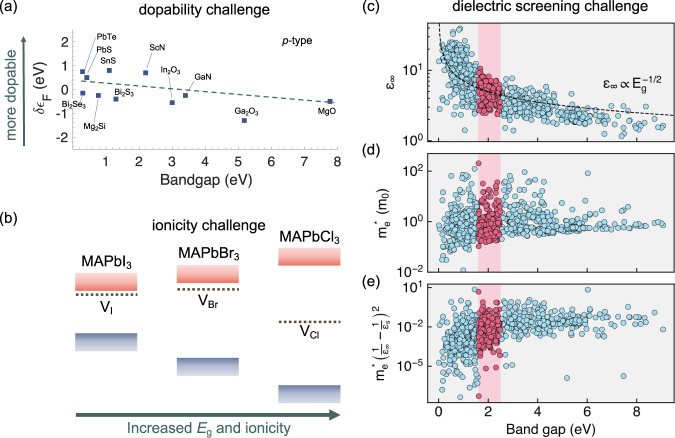
The defect challenge for wide-gap semiconductors. **a** Dopability challenge. Plot of the ability to dope materials (δε_F_) as a function of their bandgap (*E*_g_). δε_F_ is defined as the difference between the band-edge (valence band maximum for p-type doping) and doping pinning energy. δε_F_ from defect calculations in ref. 10, while the bandgaps shown are experimentally measured values. Trend line added to guide the eye. **b** Ionicity challenge. Energy of the (0/+) transition level of the halide vacancy for CH_3_NH_3_PbX_3_ perovskites. Dielectric screening challenge. **c** Plot of electronic dielectric constant (ε_∞_), **d** electron effective mass (*m*_e_), and **e** Fröhlich polaron binding energy factor (in which ε_S_ is the static dielectric constant) against bandgap for binary compounds in the Materials Project database^20^. The shaded red regions highlight the bandgap range 1.6–2.5 eV of interest. The data for panels **c**–**e** were calculated using density functional theory within the generalised gradient approximation. Whilst bandgaps are underestimated, the underlying physical trends are expected to be valid. Effective mass and dielectric tensors were isotropically averaged.

Many materials can be doped by either electrons (n-type) or holes (p-type) but not both. Such doping asymmetry is a feature of wide-bandgap systems. One example is the field of transparent oxide electronics where n-type doping is possible (e.g., in ZnO, SnO_2_, In_2_O_3_), yet effective p-type doping is much more challenging. This type of behaviour is often rationalised by using the doping limit rules^9^ (and sometimes explained by the concept of Fermi level pinning), which state that the lower the ionisation potential (valence band energy), the easier it is to make a material p-type, and the higher the electron affinity (conduction band energy), the easier it is to make it n-type. The limit itself is imposed by the spontaneous formation of charge compensating point defects. As such, wide-gap semiconductors with low electron affinity and/or high ionisation potential generally have lower dopability to one or both carriers. This can be seen in Fig. 1a, in which there is an overall decrease in the predicted dopability (δε_F_) as the bandgap increases (especially for the case of p-type dopability shown)^10^. Whilst the correlation between band-edge position and dopability is established, the link between dopability and defect tolerance is still unresolved.

### Increased ionicity

Ionic materials typically have larger bandgaps than their covalent counterparts, which can be seen, for example, in comparing NaCl (8.5 eV) to silicon (1.12 eV), or LiF (14 eV) to diamond (5.5 eV). As charge transfer increases, a stronger Coulomb potential separates the valence and conduction bands. For example, the bandgap of CH_3_NH_3_PbI_3_ can be continuously increased from 1.59 to 3.04 eV by changing the halide species to the more electronegative Br or Cl, or using mixtures thereof (Fig. 1b)^11^. The established defect tolerance of CH_3_NH_3_PbI_3_ is not preserved across the entire halide composition range. The halide vacancy (0/+) donor level becomes deeper through the series (Fig. 1b), due to the lower electron affinity, as well as the smaller lattice constant of CH_3_NH_3_PbCl_3_ leading to greater overlap between Pb dangling bonds when halide vacancies form^12^. The electrostatic potential at the vacancy site becomes sufficiently large to localise an unpaired electron to form a deep state with large structural relaxation energy. For the case of ZnO_1–*x*_S_*x*_, a nitrogen acceptor is deep above the valence band for small values of *x*, but becomes shallower for S-rich materials^13^. Increased ionicity can lead to stronger carrier trapping and activate previously benign defects for non-radiative electron-hole recombination pathways.

### Weaker dielectric screening

As the bandgap of a material increases, the polarisability also tends to decrease. Indeed, the high-frequency dielectric constant is inversely proportional to the square-root of the bandgap (Fig. 1c). There is also a correlation with effective mass as the interaction between the valence and conduction band edges is weakened (Fig. 1d). Defect tolerance requires low charge capture cross-sections, which are strongly influenced by both the dielectric constant and effective mass of the host material. A large dielectric constant screens electrostatic interactions between charged defects and charge-carriers, and maintains a low Sommerfeld factor (see ref. 4 for further details). A low effective mass ${m}_{0}^{\ast }$ facilitates high mobilities and enhances the delocalisation of the defect wavefunctions with the band edges. These factors also control the degree of polaron formation, which in the simplified Fröhlich model gives the relation.

1 $${E}_{{{{{{\rm{p}}}}}}}\propto {m}_{0}^{\ast }{\left(\frac{1}{{\varepsilon }_{\infty }}-\frac{1}{{\varepsilon }_{{{{{{\rm{s}}}}}}}}\right)}^{2}$$

where *E*_p_ is the polaron binding energy, and *ε*_s_ and *ε*_∞_ are the static (low-frequency) and high-frequency dielectric constants, respectively. This relationship presents a challenge, as based on these considerations, wide-gap materials should have less mobile charge-carriers (as seen from the positive correlation between *E*_p_ and *E*_g_ in Fig. 1e) with higher recombination rates (due to the smaller dielectric constants and larger effective masses). Even in the case of III–V semiconductors, the exceptional 9000 cm^2^ V^−1^ s^−1^ electron mobility for GaAs (*E*_g_ = 1.5 eV, *m*_e_ = 0.07, *ε*_∞_ = 10.9) is reduced to 900 cm^2^ V^−1^ s^−1^ for GaN (*E*_g_ = 3.5 eV, *m*_e_ = 0.22, *ε*_∞_ = 4.9)^14^.

## Future opportunities

A promising avenue to enhance dielectric screening is to identify host materials with large ionic (vibrational) contributions to the static dielectric constant. This component is sensitive to the underlying structure and vibrations, which can be tuned through crystal engineering. The net ionic screening (defined as ε_s_ – ε_∞_) is decoupled from the bandgap (Supplementary Fig. 1). Instead, large ionic contributions are found in materials containing polarisable cations (such as Bi or Pb) and in materials close to a polar phase transition (such as SrTiO_3_ and other perovskite structured crystals). These factors are realised in the emerging absorber SbSI which, despite its relatively wide bandgap of 1.86 eV, possesses a giant room temperature dielectric constant >10^4^ (along the *c* axis) due to a ferroelectric Curie temperature around 291 K. This route has also recently been exploited in the development of ferroelectric oxide PVs, such as KNbO_3_–BaNi_0.5_Nb_0.5_O_3_ solid solutions^15^. Here, spontaneous electric polarisation promotes the separation of photoexcited carriers and enables the generation of above-bandgap photovoltages. As oxides typically possess larger bandgaps, this approach may prove especially fruitful for the identification of wide-gap defect-tolerant candidates.

**Fig. 2:**
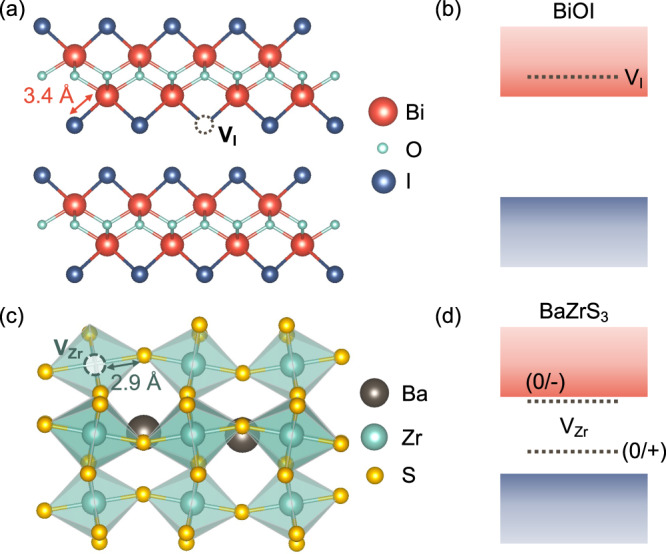
Wide-bandgap semiconductors with non-harmful traps. **a** Crystal structure and **b** band diagram of BiOI, showing the (0/+) transition level of iodide vacancies (V_I_)^16^. **c** Crystal structure and **d** band diagram of BaZrS_3_, showing the deep donor (0/+) and shallow acceptor (0/–) transition levels of zirconium vacancies (V_Zr_)^19^.

Inspiration on how to achieve shallow traps in wide-gap materials can be found in bismuth oxyiodide (BiOI) and BaZrS_3_ (*E*_g_~1.8–1.9 eV). In BiOI, the Bi-I bonds are comparatively long (3.4 Å [Fig. 2a] vs. 3.7 Å for Pb-I in CH_3_NH_3_PbI_3_)^16^. When I vacancies form, the neighbouring Bi atoms are sufficiently far apart to avoid significant orbital overlap, leading to small energy splittings in the bonding-antibonding combinations formed from the dangling bonds^17^. This, coupled with the moderate electron affinity of 4.1 eV, results in the V_I_ (0/+) donor transition level being resonant within the conduction band (Fig. 2b)^16^. The inert nature of I vacancies has been verified experimentally^18^. Preliminary defect chemistry analysis indicates that BaZrS_3_ benefits from the flexible perovskite structure (Fig. 2c), similar to the metal halides, which ‘cleans up’ deep traps from the bandgap (Fig. 2d)^19^. These recent results further motivate efforts at overcoming the challenges of growing high-quality thin films of chalcogenide perovskites at low temperatures to exploit these properties in PV devices.

In conclusion, defect tolerance has allowed select low-bandgap semiconductors to achieve efficient device performance when processed by low-temperature, cost-effective methods. As a new generation of wide-bandgap semiconductors gain increasing technological significance, overcoming their unique defect challenges is of paramount importance. Promising strategies include identifying materials with high ionic dielectric constants but weak Fröhlich coupling, translating the defect chemistry of halide perovskites to alternative chalcogenide and pnictide materials, as well as using the electron affinity and ionisation potential as indicators for the dopability of the material and whether shallow traps form. These strategies, which can be used in tandem, are generalisable across all types of point defects, and not solely the vacancies discussed above as illustrative examples. Other levers for engineering defect tolerance will include built-in and applied strain and order-disorder transitions in mixed cation and mixed anion systems. Initial work will require a detailed analysis of the defects present in these materials through characterisation and calculations in order to identify parameters that are generalisable into design rules. Addressing the defect challenge for wide-gap semiconductors offers a rich opportunity to unveil new insights into chemical, structural and electronic descriptors for defect tolerance, as well as routes to push forward these materials as a cost-effective technology for energy and healthcare applications.

## Supplementary information

Supplementary Information

## Data Availability

No new experimental data was generated in this work.

## Code availability

The data for Fig. 1c–e and Supplementary Fig. 1 were obtained from the Materials Project database^20^, and the code used to extract these data and generate the plots is available from https://gist.github.com/utf/3b06d3850a6b1d643613af3bc7cb3842.
