# Supplementary material for: The defect challenge of wide-bandgap semiconductors for photovoltaics and beyond
Source: Nat Commun. 2022 Aug 11;13:4715. doi: 10.1038/s41467-022-32131-4 (PMC9372133; doi:10.1038/s41467-022-32131-4)
Supplement: Supplementary file 1 — Supplementary Information [file 41467_2022_32131_MOESM1_ESM.pdf]

Supplementary Information for

The Defect Challenge of Wide-Bandgap Semiconductors for Photovoltaics and Beyond

Alex Ganose,[1] David O. Scanlon,[2,3] Aron Walsh,[1,4] Robert L. Z. Hoye[1,*]

[1] Department of Materials, Imperial College London, Exhibition Road, London SW7 2AZ, United Kingdom

[2] Department of Chemistry, University College London, 20 Gordon Street, London WC1H 0AJ, United Kingdom

[3] Thomas Young Centre, University College London, Gower Street, London WC1E 6BT, United Kingdom

[4] Department of Materials Science and Engineering, Yonsei University, Seoul 120-749, Republic of Korea

* Email: r.hoye@imperial.ac.uk

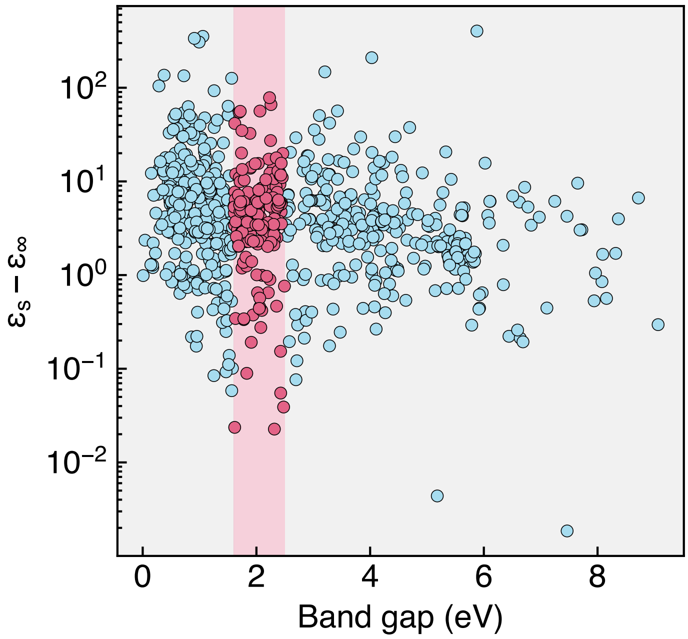

Supplementary Fig. 1. Difference between the static (ε_s) and electronic (ε_∞) dielectric constants of binary compounds against their bandgap. The red region indicates the bandgap range of interest (1.6–2.5 eV). Data obtained from Materials Project.[1]

Reference

1. Jain, A. *et al.* Commentary: The materials project: A materials genome approach to accelerating materials innovation. *APL Mater.* **1**, 011002 (2013).
